# Vandetanib (ZD6474), an inhibitor of VEGFR and EGFR signalling, as a novel molecular-targeted therapy against cholangiocarcinoma

**DOI:** 10.1038/sj.bjc.6604988

**Published:** 2009-03-24

**Authors:** D Yoshikawa, H Ojima, A Kokubu, T Ochiya, S Kasai, S Hirohashi, T Shibata

**Affiliations:** 1 Cancer Genomics Project, National Cancer Center Research Institute, Tokyo, Japan; 2 Division of Gastroenterological and General Surgery, Department of Surgery, Asahikawa Medical College, Asahikawa, Japan; 3 Pathology Division, National Cancer Center Research Institute, Tokyo, Japan; 4 Section for Studies on Metastasis, National Cancer Center Research Institute, Tokyo, Japan

**Keywords:** EGFR, VEGFR, cholangiocarcinoma, *in vivo* imaging, molecular-targeted therapy

## Abstract

Cholangiocarcinoma is an intractable cancer, with no effective therapy other than surgical resection. Elevated vascular endothelial growth factor (VEGF) and epidermal growth factor receptor (EGFR) expressions are associated with the progression of cholangiocarcinoma. We therefore examined whether inhibition of VEGFR and EGFR could be a potential therapeutic target for cholangiocarcinoma. Vandetanib (ZD6474, ZACTIMA), a VEGFR-2/EGFR inhibitor, was evaluated. Four human cholangiocarcinoma cell lines were molecularly characterised and investigated for their response to vandetanib. *In vitro*, two cell lines (OZ and HuCCT1), both of which harboured *KRAS* mutation, were refractory to vandetanib, one cell line (TGBC24TKB) was somewhat resistant, and another cell line (TKKK) was sensitive. The most sensitive cell line (TKKK) had *EGFR* amplification. Vandetanib significantly inhibited the growth of TKKK xenografts at doses ⩾12.5 mg kg^−1^ day^−1^ (*P*<0.05), but higher doses (50 mg kg^−1^ day^−1^, *P*<0.05) of vandetanib were required to inhibit the growth of OZ xenografts. Vandetanib (25 mg kg^−1^ day^−1^) also significantly (*P*=0.006) prolonged the time to metastasis in an intravenous model of TKKK metastasis. Inhibiting both VEGFR and EGFR signalling appears a promising therapeutic approach for cholangiocarcinoma. The absence of *KRAS* mutation and the presence of *EGFR* amplification may be potential predictive molecular marker of sensitivity to EGFR-targeted therapy in cholangiocarcinoma.

Cholangiocarcinoma (cancer of the bile duct epithelium) is one of the intractable cancers, whose incidence and mortality rates, especially those of intrahepatic cholangiocarcinoma (IHCC), are increasing worldwide (Khan *et al*, 2005). As cholangiocarcinoma is difficult to diagnose at an early stage and no effective therapy other than complete resection has been established, its prognosis is very poor (5-year survival is 0–40% even in resected cases) (Khan *et al*, 2005; Sirica, 2005). Although gemcitabine-based chemotherapy regimens have shown some potential in the treatment of cholangiocarcinoma in recent years (Knox *et al*, 2005), novel therapeutic strategies are required.

Recently, molecular-targeted therapies have become available and have shown clinical benefit in some cancers (Gonzalez Angulo *et al*, 2006; Tabernero, 2007). Epidermal growth factor receptor (EGFR) and vascular endothelial growth factor receptor (VEGFR) have emerged as potential therapeutic targets in cholangiocarcinoma. Several studies have shown overexpression of EGFR, amplification and mutation of *EGFR* genes (Gwak *et al*, 2005; Nakazawa *et al*, 2005; Leone *et al*, 2006), and overexpression of VEGF protein (Tang *et al*, 2006) in cholangiocarcinoma. A phase II study of erlotinib, an EGFR kinase inhibitor, for advanced cholangiocarcinoma suggests the clinical benefit for EGFR inhibition in patients with cholangiocarcinoma (Philip *et al*, 2006).

The EGFR signalling pathway is associated with the progression, proliferation, migration, and survival of cancer cells (Yarden and Sliwkowski, 2001), and VEGF plays a key role in tumour-associated neo-angiogenesis, which provides a tumour with oxygen, nutrition, and a route for metastasis (Tabernero, 2007). In addition, VEGF upregulation in tumour cells is considered to be a mechanism of resistance to EGFR inhibitors (Viloria Petit *et al*, 2001). Earlier, we have also reported that EGFR and VEGF overexpressions are frequent in cholangiocarcinoma (∼20 and 50%, respectively), that EGFR overexpression is an independent prognostic factor in IHCC, and that VEGF expression is associated with intrahepatic metastasis in IHCC (Yoshikawa *et al*, 2008). These observations prompted us to hypothesise that dual inhibition of both EGFR and VEGFR may exert a synergistic anti-tumour effect in cholangiocarcinoma.

*In vivo* imaging using bioluminescence can monitor tumour growth in animals, providing longitudinal and temporal information. Its value in the assessment of anti-cancer agents *in vivo* has been recently confirmed in some animal models of cancer (Jenkins *et al*, 2003; Nogawa *et al*, 2005). In this study, we established bioluminescent cholangiocarcinoma cells and mouse xenograft models of cholangiocarcinoma, and used these to assess the activity of vandetanib (ZD6474, ZACTIMA), a VEGFR-2 and an EGFR tyrosine kinase inhibitor, using an *in vivo* imaging system.

## Materials and methods

### Cholangiocarcinoma cell lines

Four human cholangiocarcinoma cell lines derived from Japanese patients (TKKK, OZ, TGBC24TKB, and HuCCT1) were purchased from RIKEN Bio Resource Center (Tsukuba, Japan, http://www.brc.riken.jp/lab/cell/) or from the Japanese Collection of Research Bioresources (Osaka, Japan, http://cellbank.nibio.go.jp/). The TKKK cell line was derived from IHCC, and the OZ, TGBC24TKB, and HuCCT1 cell lines from extrahepatic cholangiocarcinoma.

### Subcutaneous xenograft model

All animal experiment protocols were approved by the Committee for Ethics in Animal Experimentation, and the experiments were conducted in accordance with the Guideline for Animal Experiments of the National Cancer Center (Tokyo, Japan).

Eight-week-old female BALB/c-nu/nu athymic mice were purchased from Japan SLC (Hamamatsu, Japan). A total of 8 × 10^6^ cells were suspended in 0.2 ml of culture medium without foetal bovine serum and injected subcutaneously into the right flank of the mice. Tumour volume was calculated using the following formula: (short diameter)^2^ × (long diameter)/2.

### RT–PCR analysis for EGFR, VEGF, and VEGFR-2

Total RNA and genomic DNA were extracted from the four cell lines. Total RNA of 1 *μ*g was converted into cDNA using a Transcriptor First Strand cDNA Synthesis Kit (Roche, Basel, Switzerland) in accordance with the manufacturer's instructions. mRNA expression of EGFR, VEGF, and VEGFR-2 was assessed by the RT–PCR method. Quantitative real-time PCR was conducted using LightCycler480 (Roche) in accordance with the manufacturer's instructions. TaqMan Probes (Applied Biosystems, Foster City, CA, USA) for EGFR and VEGF were used. For standardisation of the amount of RNA, expression of glyceraldehyde-3-phosphate dehydrogenase in each sample was quantified. Primers are shown in the Supplementary Table 1.

### Mutation analysis of the *EGFR* and *KRAS* genes

For the sequence analysis of *EGFR* and *KRAS*, cDNA or genomic DNA was sequenced after PCR amplification. Direct sequencing was conducted using a BigDye Terminator v3.1 Cycle Sequencing Kit (Applied Biosystems) and analysed on an ABI Prism 3100 Sequencer (Applied Biosystems). Primers are shown in the Supplementary Table 1.

### Immunohistochemistry

Tissue preparation and immunohistochemistry (IHC) were conducted as reported earlier (Yoshikawa *et al*, 2008). A polymer-based method (Envision+Dual Link System-HRP; Dako, Glostrup, Denmark) was used for EGFR, VEGF, and Ki67 staining, and a standard ABC method (Vectastain Elite ABC kit; Vector Laboratories, Burlingame, CA, USA) was used for CD34 staining. Sources and dilutions of primary antibodies were as follows: mouse anti-human EGFR (1 : 100 dilution, clone 31G7; Zymed, South San Francisco, CA, USA), rabbit anti-human VEGF (1 : 50 dilution; Zymed), mouse anti-human Ki67 (1 : 100 dilution, clone Ki-S5; Chemicon, Temecula, CA, USA), and rat anti-mouse CD34 (1 : 50 dilution, clone MEC14.7; Abcam, Cambridge, UK).

Terminal deoxynucleotidyl transferase-mediated deoxyuridine triphosphate-biotin nick end labelling (TUNEL) was conducted to assess the degree of apoptosis by using an *In Situ* Cell Death Detection Kit, POD (Roche) in accordance with the manufacturer's instructions.

### Fluorescence *in situ* hybridisation for the *EGFR* gene locus

*EGFR* gene copy number per cell was investigated by fluorescence *in situ* hybridisation (FISH) using the LSI EGFR SpectrumOrange/CEP7 SpectrumGreen probe (Vysis, Downers Grove, IL, USA), in accordance with a published protocol (Ooi *et al*, 2004). Positivity for gene amplification was defined as the presence of clustered signals or ⩾4 copies of orange signals.

### Drug and formulation

Vandetanib was provided by AstraZeneca (Macclesfield, UK). For the *in vitro* study, vandetanib was formulated as a 10-mM stock in 100% dimethylsulphoxide and stored at −20°C. Just before *in vitro* use, the stock solution was diluted in culture medium to the required concentration. For the *in vivo* study, vandetanib was administered as a homogeneous suspension with 1% polysorbate (Tween 80; MP Biomedicals, Solon, OH, USA) and administered orally once a day at 0.1 ml/10 g body weight (b.w.).

### Cell proliferation assay

Cell sensitivity to vandetanib was estimated using the 3-(4,5-dimethylthiazol-2-yl)-5-(3-carboxymethoxyphenyl)-2-(4-sulphophenyl)-2*H*-tetrazolium, inner salt (MTS) assay. The CellTiter 96 AQ_ueous_ One Solution Reagent (Promega, Madison, WI, USA) was used in accordance with manufacturer's instructions. A total of 5000 cells suspended in 100 *μ*l of 10% foetal bovine serum-containing culture medium per well were placed on a 96-well culture plate and treated with various concentrations of vandetanib (0–100 *μ*M). After 72 h, 20 *μ*l of the reagent was added, and the absorbance at 490 nm was recorded. The experiment was conducted in triplicate and repeated three times. All data were calculated as a ratio to control, which means a ratio of absorbance in each concentration of vandetanib treatment relative to that in the negative control, and presented as mean±s.d.

### Western blot analysis investigating molecular effects of vandetanib *in vitro*

Each cell starved for 24 h was exposed to various concentrations of vandetanib for 2 h, and stimulated by human EGF (1 ng ml^−1^, Wakunaga Pharmaceutical Co., Osaka, Japan) for 10 min. Cell pellets were dissolved in lysis buffer (1% Triton X-100; 10 mM Tris-HCl, pH 7.5; 150 mM NaCl) with a protease inhibitor cocktail (Roche) and a phosphatase inhibitor cocktail (Nacarai Tesque, Kyoto, Japan). Equal amounts (16 *μ*g) of cell extracts were electrophoresed, transferred to polyvinylidene difluoride membrane (Millipore, Billerica, MA, USA), and immunoblotted with the following antibodies: mouse anti-EGFR antibody (clone 13/EGFR, BD Bioscience, Franklin Lakes, NJ, USA), mouse anti-phosphorylated EGFR (pEGFR, Tyr 1068, clone 1H12; Cell Signaling Technology, Beverly, MA, USA), mouse anti-AKT (clone 2H10, Cell Signaling Technology), mouse anti-phosphorylated AKT (pAKT, Ser473, clone 587F11; Cell Signaling Technology), rabbit anti-MAPK (mitogen-activated protein kinase; Cell Signaling Technology), mouse anti-phosphorylated MAPK (Thr202/Tyr204, clone E10; Cell Signaling Technology), rabbit anti-VEGF (Lab Vision, Fremont, CA, USA), and mouse anti-β-actin (clone AC-15, Sigma, St Louis, MO, USA). All antibodies were diluted to use in accordance with the manufacturer's instructions.

### Reporter gene labelling of tumour cells

TKKK and OZ cells were transfected with a complex of 4 *μ*g pEGFP-Luc plasmid DNA (Minakuchi *et al*, 2004) and 10 *μ*l Lipofectamine 2000 reagent (Invitrogen, Carlsbad, CA, USA) in accordance with the manufacturer's instructions. Stable transfectants were selected in 200 *μ*g ml^−1^ geneticin (Invitrogen). Clones strongly expressing the luciferase gene (named TKKK-Luc and OZ-Luc) were selected and used in the *in vivo* study.

### *In vivo* tumour imaging

For the *in vivo* tumour imaging, D-luciferin 150 mg/kg per b.w. (Promega) was administered to mice by intraperitoneal injection. After 15 min, photons from animal whole bodies were counted using the IVIS imaging system (Xenogen, Alameda, CA, USA) in accordance with the manufacturer's instructions. Data were analysed using the LIVINGIMAGE 2.50.1 software (Xenogen).

### Effects of vandetanib in a xenograft model

The therapeutic and anti-metastatic activities of vandetanib were estimated using a mouse xenograft model. According to the therapeutic protocol, 8 × 10^6^ of TKKK-Luc and OZ-Luc cells were injected subcutaneously. When tumour volume exceeds 20 mm^3^, the mice were randomly divided into four treatment groups, namely vandetanib 50, 25, or 12.5 mg/kg per b.w. per day, or vehicle control. Treatment started from the next day and continued for at least 4 weeks. Photons from animal whole bodies were counted twice a week. All mice were killed at the end of the study period and subcutaneous tumours were removed completely. After the tumour volume was calculated, tumours were cut through the maximum diameter. Half of them were fixed in 10% formalin, and paraffin-embedded, and haematoxylin–eosin staining, IHC for CD34 (microvessel marker) and Ki67 (proliferation marker), and TUNEL (apoptosis marker) were conducted to investigate histological effects of vandetanib. Haematoxylin–eosin sections were observed microscopically and whole-scanned using a film scanner (Cool Scan; Nikon, Tokyo, Japan). The total tumour area and the necrotic tumour area through the maximum diameter were calculated using Image J software (NIH, http://rsb.info.nih.gov/ij/), and the percentage of the necrotic area was calculated. Evaluation of IHC for CD34 and Ki67 and for TUNEL was conducted by DY and two pathologists (HO and TS), using standard light microscopy without knowledge of any therapeutic intervention. Microvessel density (MVD) was defined as the mean number of microvessels in three fields (original magnification, × 200) containing high levels of CD34-stained microvessels (‘hotspots’). Ki67 proliferation index (PI) and apoptotic index (AI) were defined as the percentage of positive cells among 1000 tumour cells or over at the hotspot. The others were immediately frozen in liquid nitrogen and dissolved in lysis buffer with protease and phosphatase inhibitors to investigate molecular effects of vandetanib, and the expression of EGFR, pEGFR, and VEGFR-2 in both treatment (vandetanib 50 mg/kg per b.w. per day) and vehicle control groups were assessed by using western blot analysis. Rabbit anti-VEGFR-2 antibody (Lab Vision) was used in accordance with the manufacturer's instructions.

To evaluate the effects on tumour metastasis, an intravenous tumour cell-seeding model was used (anti-metastatic protocol). TKKK-Luc cells (4 × 10^6^) were suspended in 200 *μ*l of PBS and were injected into mice through the tail vein after 7 days of daily administration of vandetanib 25 mg/kg per b.w. per day or vehicle control. Mice were then treated 5 days a week for 3 months, and photon counting was conducted once a week. If the photon signal was visualised on the IVIS imaging system, the mouse was considered as having a metastasis. The time to metastasis was estimated as an index of the anti-metastatic effect of vandetanib. At the end of the study, all mice were killed and autopsied. All organs, including the lung and brain, were formalin-fixed and sliced at 3-mm intervals, and the presence of tumours was confirmed microscopically.

### Correlations between expression and gene amplification of *EGFR* in clinical samples

Epidermal growth factor receptor expression was assessed by IHC in samples from 90 cases of cholangiocarcinoma that had been resected at the National Cancer Center Hospital. Among these samples, *EGFR* gene amplification was also examined in 19 EGFR-positive and 15 EGFR-negative samples, and the correlation between protein expression and gene amplification of *EGFR* was investigated. This study was approved by the Ethics Committee of the National Cancer Center (Tokyo, Japan), and written informed consent was obtained from all patients.

### Statistics

All statistical analyses were performed with the Statview 5.0 statistical software package (Abacus Concepts, Berkeley, CA, USA). For the therapeutic protocol, change of photon count was estimated using repeated measures analysis of variance (ANOVA) followed by Dunnett's *post hoc* test. Between-group comparisons of response to vandetanib (tumour volume, necrotic area, MVD, PI, and AI) were estimated using one-way ANOVA followed by Dunnett's *post hoc* test. For the anti-metastatic protocol, the time to metastasis curve was calculated using the Kaplan–Meier method, and log-rank test was performed for the comparison of the time to metastasis curves. Correlations between treatment and occurrence of metastasis in the anti-metastatic protocol, and between expression and gene amplification of EGFR in the clinical samples were assessed using Fisher's exact probability test. All numerical data were presented as mean±s.d. Differences at *P*<0.05 were considered as statistically significant.

## Results

### Molecular characteristics of four cholangiocarcinoma cell lines

**Figure 1:**
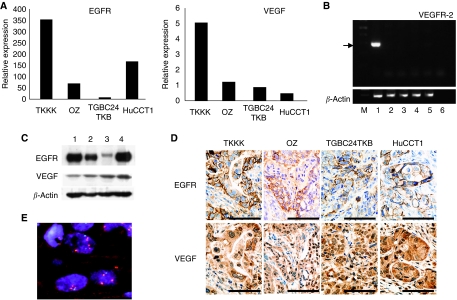
Characteristics of the four cholangiocarcinoma cells. (**A**) Epidermal growth factor receptor (EGFR) and VEGF mRNA expressions (by real-time PCR). (**B**) Vascular endothelial growth factor receptor-2 mRNA expression (VEGFR-2, by RT–PCR; lane 1, human liver tissue; lane 2, TKKK; lane 3, OZ; lane 4, TGBC24TKB; lane 5, HuCCT1; and lane 6, no RNA). The arrow indicates the band of VEGFR-2. (**C**) Epidermal growth factor receptor and VEGF expressions (by western blotting; lane 1, TKKK; lane 2, OZ; lane 3, TGBC24TKB; and lane 4, HuCCT1). (**D**) Epidermal growth factor receptor and VEGF expressions *in vivo* (by immunohistochemistry). Scale bar=0.1 mm. Epidermal growth factor receptor and VEGF were expressed in all cells, but VEGFR-2 was not. (**E**) Fluorescence *in situ* hybridization for the *EGFR* locus in TKKK (orange, EGFR; green, centrosome7).

Epidermal growth factor receptor and VEGF mRNA were detected in all four cholangiocarcinoma cell lines (Figure 1A), but VEGFR-2 mRNA was not expressed in any of them (Figure 1B). As none of these cell lines expressed VEGFR-2, we assume that the direct effect of vandetanib against these cells was mainly mediated by its anti-EGFR effect. Among the four cell lines, TKKK cells showed the highest expression of both EGFR and VEGF (Figure 1A). Epidermal growth factor receptor and VEGF proteins were also detected in all cell lines, and EGFR protein expression levels were correlated with mRNA levels, but VEGF were not (Figures 1A and C). The expression levels of VEGF mRNA may not always correspond with those of VEGF protein, as VEGF mRNA is labile under the normal oxygen tension and some translational regulation of VEGF expression has been reported (Levy *et al*, 1996; Mezquita *et al*, 2005). Epidermal growth factor receptor and VEGF protein expressions were also confirmed by IHC in xenograft tumours (Figure 1D). Fluorescence *in situ* hybridisation analysis revealed *EGFR* gene amplification only in TKKK cells (Figure 1E). We also sequenced the kinase domain of *EGFR* gene, but found no *EGFR* mutation in any of the cell lines. *KRAS* mutation was detected in OZ (Q61L) and HuCCT1 (G12D) cell lines, but not in TKKK and TGBC24TKB cell lines.

### Anti-proliferative effect of vandetanib *in vitro*

**Figure 2:**
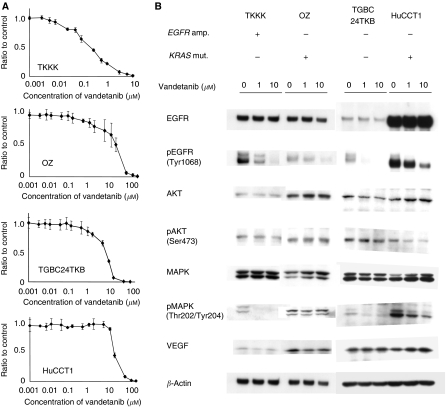
(**A**) The anti-proliferative effect of vandetanib against cholangiocarcinoma cell lines (TKKK, OZ, TGBC24TKB, and HuCCT1) *in vitro*. All data are presented as mean±s.d. A ratio to control means a ratio of absorbance in each concentration of vandetanib treatment relative to that in the negative control. (**B**) The western blot analysis of VEGF, EGFR, and phosphorylation of markers downstream of EGFR. Vandetanib inhibited phosphorylation of EGFR in all cell lines, but phosphorylation of MAPK was inhibited only in TKKK and TGBC24TKB cell lines.

The effect of vandetanib on proliferation in each cell line is shown in Figure 2A. The vandetanib IC_50_ for the PC-9 lung cancer cell line, which is sensitive to EGFR inhibitors, was reported earlier to be 0.14 *μ*M (Taguchi *et al*, 2004). Compared with this data, TKKK cells were also sensitive to vandetanib (IC_50_: 0.22 *μ*M), TGBC24TKB was moderately resistant (IC_50_: 4.5 *μ*M), and OZ and HuCCT1 cells (IC_50_s of 12.2 and 10 *μ*M, respectively) were considered refractory. Next, we examined the expression of VEGF and EGFR, and also the phosphorylation status of downstream molecules (AKT and MAPK) of EGFR in four cell lines (Figure 2B). No significant change in VEGF, EGFR, AKT, pAKT, or MAPK expression was observed after vandetanib treatment. Phosphorylation of EGFR was inhibited by vandetanib treatment in all cell lines, and it can be noted that phosphorylation of MAPK was inhibited in TKKK and TGBC24TKB (not refractory to vandetanib) cell lines but not in OZ and HuCCT1 (refractory to vandetanib) cell lines.

### Anti-tumour effects of vandetanib *in vivo*

**Figure 3:**
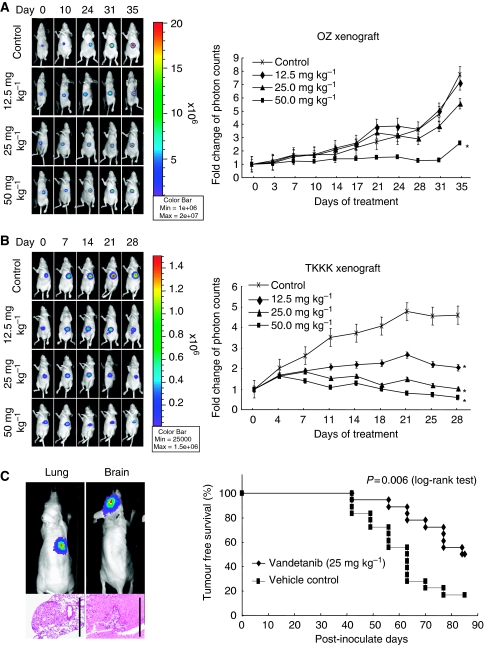
The anti-tumour effects of vandetanib *in vivo* in (**A**) OZ-Luc xenografts (*n*=7) and (**B**) TKKK-Luc xenografts (*n*=10). All data are presented as mean±s.d. ^*^P<0.05 *vs* control group (repeated measures ANOVA and Dunnett's test). (**C**) The anti-metastatic effects of vandetanib. Time to occurrence of TKKK-Luc metastasis was significantly prolonged by vandetanib (*P*=0.006, log-rank test). Metastases were found in lung or brain and histologically confirmed as micro-metastasis. Scale bar=1 mm.

**Figure 4:**
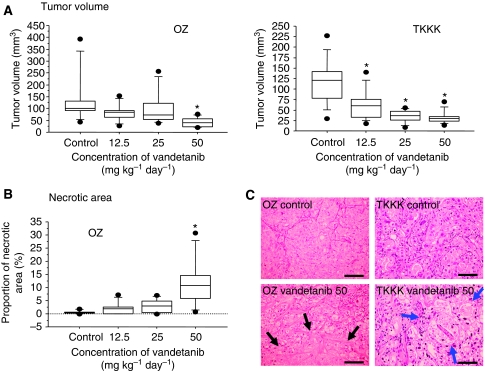
Tumour volume at the end of the therapeutic protocol in (**A**) OZ-Luc xenografts (*n*=7) and TKKK-Luc xenografts (*n*=10). (**B**) The proportion of the necrotic area in the OZ-Luc xenografts. ^*^P<0.05 *vs* control group (ANOVA and Dunnett's test). Mean values of each index are shown in Supplementary Table 2. (**C**) Haematoxylin–eosin staining for removed tumours. Massive necrosis was found in the vandetanib-treated OZ xenografts (black arrows), and tumour glands, including degenerate tissues, were increased in the vandetanib-treated TKKK xenografts (blue arrows). Scale bar=0.1mm.

**Figure 5:**
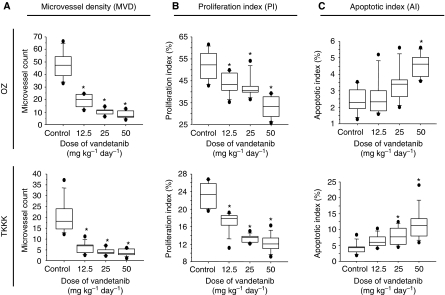
(**A**) Microvessel density (MVD), (**B**) proliferation index (PI), and (**C**) apoptotic index (AI) of removed tumours. ^*^P<0.05 *vs* control group (ANOVA and Dunnett's test). Mean values of each index are shown in Supplementary Table 2.

**Figure 6:**
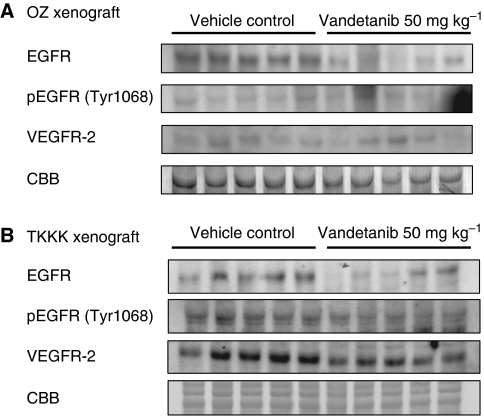
The western blot analysis of EGFR, pEGFR, and VEGFR-2 expressions after vandetanib (50 mg/kg per b.w.) and vehicle treatment in the therapeutic protocol. (**A**) OZ-Luc xenografts and (**B**) TKKK-Luc xenografts. CBB, Coomassie Brilliant Blue staining.

The *in vivo* anti-tumour effect of vandetanib against an *in vitro*-sensitive cell line (TKKK-Luc derived from TKKK) and an *in vitro* refractory cell line (OZ-Luc derived from OZ) was then evaluated using subcutaneous xenografts. The tumour growth curves analysed by the IVIS imaging system are shown in Figures 3A and B. As expected from the *in vitro* study, growth of the TKKK-Luc xenograft was significantly suppressed by vandetanib treatment at a lower dose, 12.5–25 mg kg^−1^, whereas reduction of the OZ-Luc xenograft tumour was observed at a vandetanib dose of 50 mg kg^−1^. At the end of the study, tumour volume was significantly lower in the vandetanib 50 mg kg^−1^ group of the OZ-Luc xenograft and in the 12.5–50 mg kg^−1^ group of the TKKK-Luc xenograft than in the vehicle-treated control group (Figure 4A). Histologically, OZ-Luc tumours treated with vandetanib showed substantial necrosis, the necrotic area being significantly greater than for the vehicle-treated control group (Figures 4B and C, black allows). TKKK-Luc tumours did not show any substantial necrotic area in either the vandetanib-treated or the vehicle-treated groups (data not shown), but tumour glands including degenerate tissues were increased (Figure 4C, blue arrows). Feature of cytological degeneration such as clear cytoplasmic change were frequent in the vandetanib-treated TKKK and OZ xenografts compared with the vehicle-treated tumours (Figure 4C). Microvessel density (Figure 5A) and PI (Figure 5B) were significantly decreased in all vandetanib-treated groups of both the OZ-Luc and TKKK-Luc xenografts. AI (Figure 5C) was significantly increased in the vandetanib 50 mg kg^−1^ group of the OZ-Luc xenograft, and in the vandetanib 25 and 50 mg kg^−1^ groups of TKKK-Luc xenograft. In both xenografts, EGFR, pEGFR, and VEGFR-2 expression were reduced by vandetanib treatment (Figures 6A and B). According to the molecular characters of these cells, VEGFR-2 is supposed to be expressed in the tumour stroma, but not in tumour cells.

Box plots in Figures 4 and 5 present that upper and under bar means 90th and 10th percentile, box means between 25th and 75th percentile, and the line in the box means median. Representative expression of CD34, Ki67, and TUNEL is shown in Supplementary Figure 1, and mean values of histological assessment data are shown in Supplementary Table 2.

### Anti-metastatic effects of vandetanib *in vivo*

We next assessed whether vandetanib treatment might have effects on the establishment and growth of distant metastasis using an intravenous tumour cell-seeding model. Preliminary evaluations confirmed the presence of lung metastases within 3 months in two of three (67%) mice inoculated with TKKK-Luc cells from the tail vein. Next, 18 mice per group (vandetanib 25 mg kg^−1^ or vehicle treatment) were challenged with intravenous tumour cell inoculation, and time to metastasis was assessed using the IVIS imaging system. No mice died throughout the 3-month observation period. The time to metastasis was significantly prolonged in the vandetanib group compared with the vehicle-treated group (median: 84 *vs* 63 days, *P*=0.006, Figure 3C). At the end of the study, metastases were present in 15 out of 18 (83.3%) of the control group and 9 out of 18 (50.0%) of the vandetanib group (*P*=0.075). Lung and brain metastases were found in 20 and 6 mice, respectively. Most tumours were histologically identified as micronodules or assembled tumour cells (Figure 3C).

### Correlations between expression and gene amplification of *EGFR* in clinical samples

Out of 90 cholangiocarcinoma samples, 19 (21.1%) were tested EGFR-positive by IHC. Among these cases, *EGFR* gene amplification was examined by FISH in the 19 EGFR-positive and 15 EGFR-negative samples. Of these 34 samples 8 had *EGFR* gene amplification, of which all 8 were confirmed as EGFR-positive by IHC. In contrast, none of the EGFR-negative samples were found to have gene amplification (*P*=0.0045).

## Discussion

Vandetanib is a tyrosine kinase inhibitor of both VEGFR-2 and EGFR, and preclinical studies have confirmed its anti-tumour effects in a range of cancer types (Wedge *et al*, 2002; Ciardiello *et al*, 2004; Taguchi *et al*, 2004; Williams *et al*, 2004; Yano *et al*, 2005). Phase III clinical studies are now underway with vandetanib in non-small-cell lung cancer following promising results in phase I and II studies (Holden *et al*, 2005; Natale *et al*, 2006; Tamura *et al*, 2006; Heymach *et al*, 2007a, 2007b). We have reported earlier that both EGFR and VEGF overexpressions are associated with progression of cholangiocarcinoma (Yoshikawa *et al*, 2008), and hypothesised that simultaneously blocking the EGFR and VEGF pathways might have synergistic therapeutic effects against cholangiocarcinoma. In this study, we investigated the efficacy of vandetanib in cholangiocarcinoma cell lines and in xenograft models, and report here that vandetanib strongly inhibits tumour progression *in vivo*.

### Anti-proliferative effects of vandetanib *in vitro*

As VEGFR-2 was not expressed in any of cholangiocarcinoma cell lines, we assumed that the anti-proliferative effects of vandetanib observed in this *in vitro* study were mainly because of the inhibition of EGFR signalling. All cholangiocarcinoma cell lines examined in this study expressed EGFR and VEGF, but the degree of the anti-proliferative effect of vandetanib *in vitro* varied between the cell lines. TKKK cells were sensitive to vandetanib, TGBC24TKB cells were moderately resistant to vandetanib, whereas OZ and HuCCT1 cells were refractory to vandetanib. This finding is partly consistent with an earlier report that HuCCT1 cell line was resistant to the EGFR inhibitor, erlotinib (Jimeno *et al*, 2005). It is interesting that *KRAS* mutations were found in both cell lines (HuCCT1 and OZ) considered refractory to vandetanib in this study, and *KRAS* mutation has been reported as a mechanism of resistance to EGFR inhibitors in lung and colorectal cancers (Pao *et al*, 2005; Lievre *et al*, 2006). Vandetanib strongly suppressed EGFR phosphorylation in this study, but phosphorylation of downstream MAPK was not inhibited in the OZ and HuCCT1 cell lines. These *in vitro* results suggest that in cholangiocarcinoma cells, upregulation of the RAS/RAF/MAPK pathway by mutant *KRAS* might counteract the anti-growth effect of vandetanib by EGFR inhibition. The incidence of *KRAS* mutation in cholangiocarcinoma is estimated to be 54–67% (Tada *et al*, 1990; Tannapfel *et al*, 2000), and therefore it may be important to examine the *KRAS* status when evaluating the activity of EGFR inhibitors in cholangiocarcinoma.

In non-small-cell lung cancer, *EGFR* mutation and/or amplification have been reported as possible predictive factors of sensitivity to EGFR tyrosine kinase inhibitors (Lynch *et al*, 2004; Paez *et al*, 2004; Pao *et al*, 2004; Cappuzzo *et al*, 2005). Of the cell lines without *KRAS* mutation, TKKK, which has *EGFR* amplification, was most sensitive to vandetanib. The incidence of *EGFR* mutations in cholangiocarcinoma is reported as 13.6–15.0% (Gwak *et al*, 2005; Leone *et al*, 2006). However, we did not detect mutation in the kinase domain of the *EGFR* gene in the cell lines used in this study. We have reported earlier that EGFR overexpression occurs in ∼20% of primary cholangiocarcinomas and is associated with tumour progression and poor outcome (Yoshikawa *et al*, 2008). In this study, our FISH analysis of clinical samples revealed that *EGFR* gene amplification was present in 42% (8 out of 19) of samples with EGFR overexpression, but absent in samples lacking EGFR overexpression. This result is consistent with an earlier report that *EGFR* amplification was found in 6.8% of cholangiocarcinomas (Nakazawa *et al*, 2005). Collectively, the *EGFR* and *KRAS* gene status may be a potential biomarker for predicting the response to inhibitors of EGFR including vandetanib in cholangiocarcinoma.

### Anti-tumour effects of vandetanib *in vivo*

On the basis of the *in vitro* data, we tested TKKK (the most sensitive) and OZ (the most resistant) cells in an *in vivo* therapeutic model. As VEGFR-2 was not expressed in both cells, direct anti-tumour effect of vandetanib in this study was anti-EGFR inhibition, and anti-VEGFR-2 inhibition of vandetanib was exerted in the tumour stroma. Vandetanib greatly suppressed the tumour growth of the TKKK xenograft through anti-EGFR and VEGFR-2 inhibition, consistent with the *in vitro* study. However, vandetanib also inhibited tumour growth even in the OZ (refractory to EGFR inhibition) xenograft, when given at higher dose. Vascular endothelial growth factor receptor-2 expression in OZ xenograft with high-dose vandetanib treatment was also reduced, and histologically, both TKKK and OZ tumours treated with vandetanib showed necrosis, reduced microvessels, reduced proliferation, and increased apoptosis. Therefore, the anti-tumour effect of vandetanib in this model appears to be mediated by inhibiting tumour angiogenesis (anti-VEGFR effect) as well as by directly inhibiting tumour cell proliferation (anti-EGFR effect). However, at lower doses of vandetanib, growth of OZ (refractory to the anti-proliferative effects of EGFR inhibition) xenograft was not significantly inhibited despite the proliferation and angiogenesis being reduced. These *in vivo* experiments suggest that anti-EGFR treatment is effective in cholangiocarcinoma with activated EGFR signalling (e.g., *EGFR* amplification), and that inhibiting stromal angiogenesis through VEGFR inhibition also contributes to abrogate tumour environment and suppress tumour growth, although the synergistic effect between EGFR and VEGFR-2 inhibition was not clear in this study. As cholangiocarcinoma cases expressing VEGFR-2 was reported (Wiedmann *et al*, 2006), VEGFR-2 inhibition may also be directly effective in a part of cholangiocarcinoma. Collectively, targeting both angiogenesis and the active growth signal pathway, for example, inhibiting EGFR, might exert an auxiliary effect, leading to robust tumour regression in cholangiocarcinoma.

### Anti-metastatic effects of vandetanib *in vivo*

Metastasis is a main cause of cancer death, and intrahepatic or lymph node metastases are independent prognostic factors in cholangiocarcinoma (Yoshikawa *et al*, 2008). An *in vivo* imaging system was used in the intravenous tumour cell-seeding study to elucidate whether vandetanib has an anti-metastatic effect. As *in vivo* tumour imaging can observe chronological changes in tumour growth in the individual animals with a high degree of sensitivity, which has been difficult to estimate by other current methods (Jenkins *et al*, 2003), time to metastasis was assessed as an index of anti-metastatic effects in our model. The time to metastasis in the vandetanib-treated group was significantly longer than that in the vehicle-treated group, although the final incidence of metastasis was not statistically different between the two groups at the end of study.

Decreasing activity of MAPK, which is a downstream molecule of the EGFR pathway, reduces tumour proliferation *in vivo* (Aguirre Ghiso *et al*, 2003). In animal models, an EGFR inhibitor, gefitinib, prevents carcinogenesis of gallbladder and lung cancer (Kiguchi *et al*, 2005; Yan *et al*, 2006), and reduces the incidence of metastasis of prostate carcinoma cells (Angelucci *et al*, 2006). In our model, EGFR inhibition contributed to the anti-metastatic effect of vandetanib. Moreover, angiogenesis is essential for the growth of tumours more than 1–2 mm in diameter (Fidler and Ellis, 1994; Ellis and Fidler, 1996; Yano *et al*, 2005), and VEGF is necessary for the formation of metastatic tissues at the primary site (Küsters *et al*, 2007). It is possible that VEGFR inhibition at the primary site may reduce the hematogenic metastasis in cholangiocarcinoma. Indeed, VEGF expression is associated with intrahepatic metastasis in IHCC (Yoshikawa *et al*, 2008). Although the underlying molecular mechanism remains to be clarified, our results indicate that vandetanib may have potential as a postoperative adjuvant therapy to inhibit the establishment and growth of metastases in cholangiocarcinoma.

### Conclusion

In summary, our preclinical study revealed that dual blockade of VEGFR and EGFR signalling by vandetanib resulted in considerable therapeutic effects in a mouse model of cholangiocarcinoma. Our results also suggest that vandetanib may have potential as a postoperative adjuvant therapy in these tumours. Moreover, both the absence of *KRAS* mutation and the presence of *EGFR* amplification appear promising biomarkers for predicting the response of cholangiocarcinoma to agents that inhibit EGFR (such as vandetanib). As no standard chemotherapy for cholangiocarcinoma has been established to date, further investigation at the clinical setting, including biomarker evaluation, is urgently required.
