# Supplementary figures and images for: Vandetanib (ZD6474), an inhibitor of VEGFR and EGFR signalling, as a novel molecular-targeted therapy against cholangiocarcinoma
Source: Br J Cancer. 2009 Mar 24;100(8):1257–66. doi: 10.1038/sj.bjc.6604988 (PMC2676540; doi:10.1038/sj.bjc.6604988)

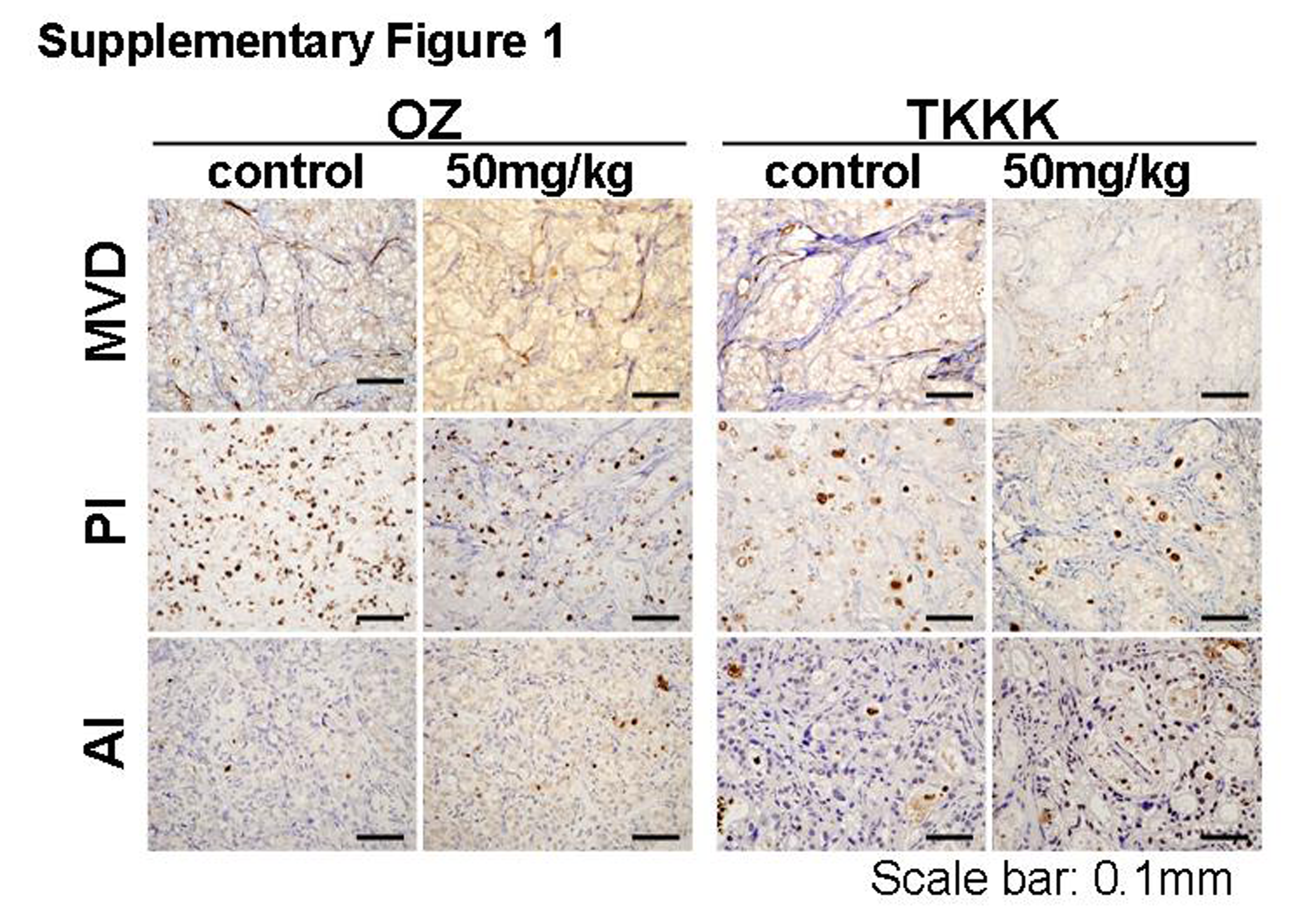

Supplement: Supplementary Figure 1 [file 6604988x1.tif]

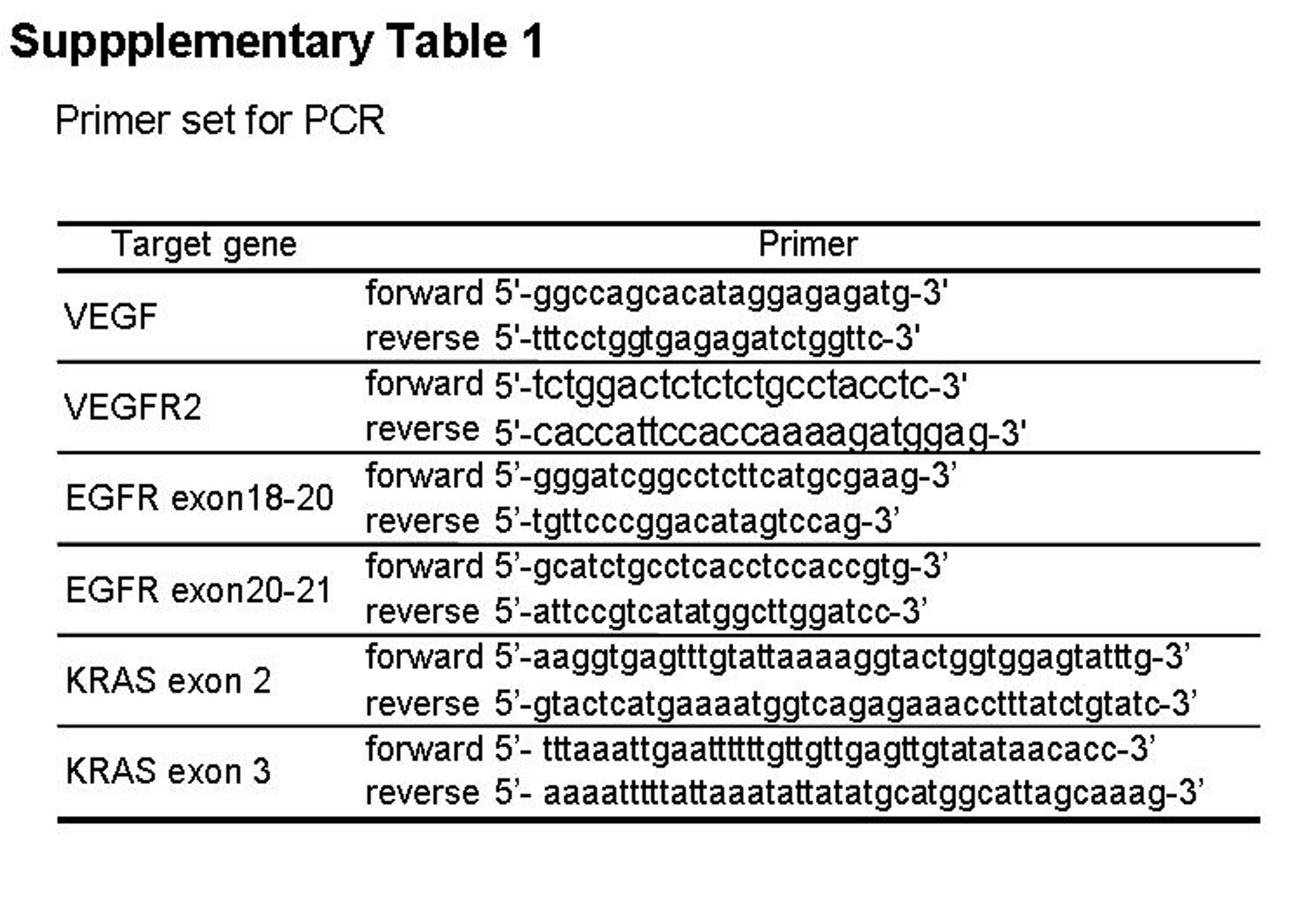

Supplement: Supplementary Table 1 [file 6604988x2.tif]

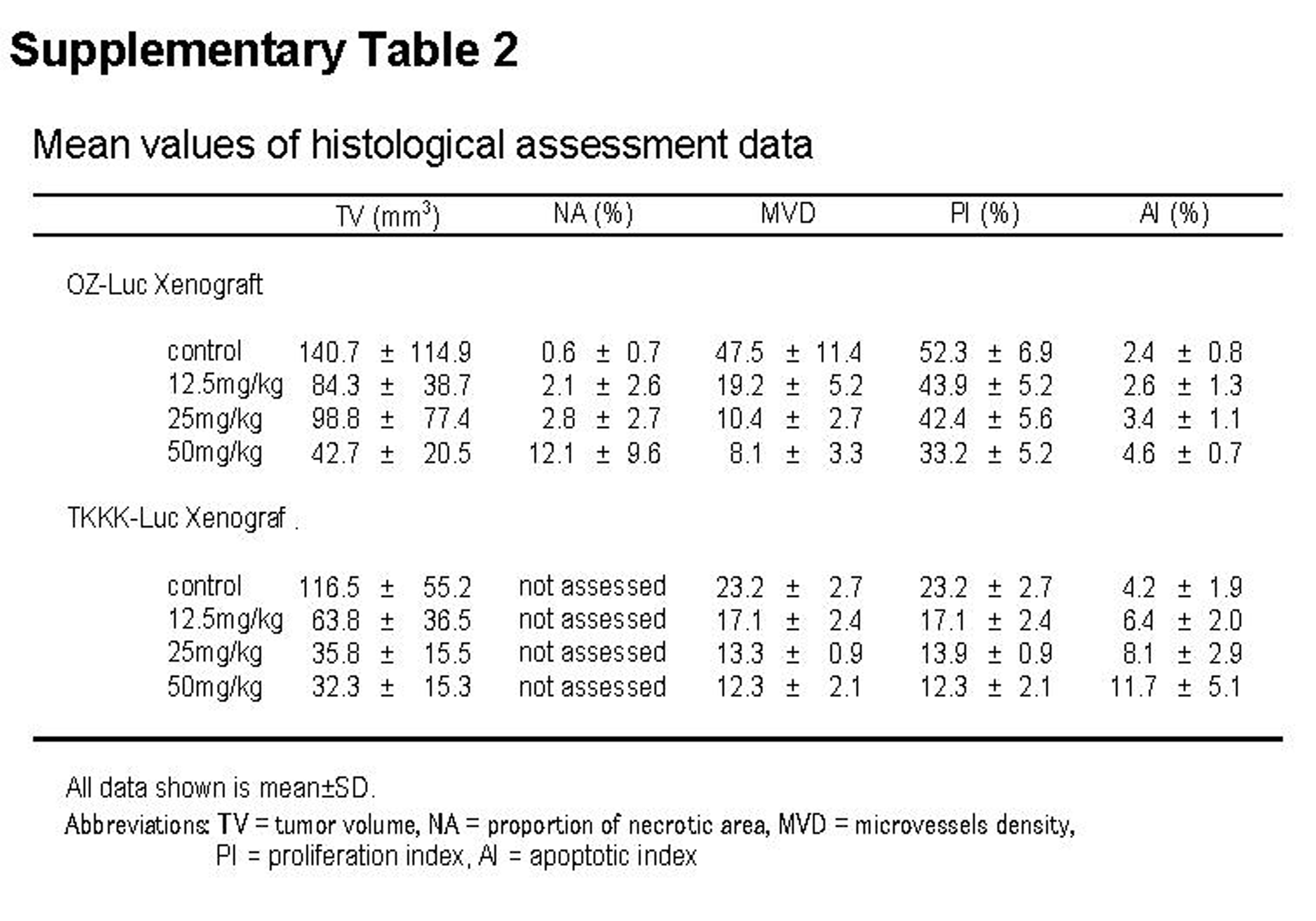

Supplement: Supplementary Table 2 [file 6604988x3.tif]
