# Supplementary material for: Vandetanib (ZD6474), an inhibitor of VEGFR and EGFR signalling, as a novel molecular-targeted therapy against cholangiocarcinoma
Source: Br J Cancer. 2009 Mar 24;100(8):1257–66. doi: 10.1038/sj.bjc.6604988 (PMC2676540; doi:10.1038/sj.bjc.6604988)
Supplement: Supplementary Figure Legend [file 6604988x4.doc]

**Legends for supplementary data**

**Supplementary Figure 1**

Expressions of CD34 (endothelial cell marker, top panels), Ki67 (proliferation marker, middle panels) and TUNEL (apoptosis marker, bottom panels) in representative tumors of subcutaneous xenograft models (left: control, right: vandetanib-treated). In vandetanib 50mg/kg/b.w. treated groups, microvessels density (MVD) and proliferation index (PI) were decreased and apoptotic index (AI) was increased compared to those of control groups in both OZ and TKKK xenografts. Scale bars indicate 100 μm.

**Supplementary Table 1**

Primer sets used in this study.

**Supplementary Table 2**

Mean values of histological assessment data about anti-tumor effects　in subcutaneous xenograft models. All data is shown as mean±SD.
